# Use of psycho‐oncological services by prostate cancer patients: A multilevel analysis

**DOI:** 10.1002/cam4.2999

**Published:** 2020-03-31

**Authors:** Clara Breidenbach, Rebecca Roth, Lena Ansmann, Simone Wesselmann, Sebastian Dieng, Ernst‐Günther Carl, Günter Feick, Alisa Oesterle, Peter Bach, Burkhard Beyer, Rainer Borowitz, Jörg Erdmann, Frank Kunath, Simba‐Joshua Oostdam, Igor Tsaur, Friedemann Zengerling, Christoph Kowalski

**Affiliations:** ^1^ German Cancer Society Berlin Germany; ^2^ Institute of Medical Statistics and Computational Biology (IMSB) Faculty of Medicine University of Cologne Koln Germany; ^3^ Organizational Health Services Research Department for Health Services Research Carl von Ossietzky University of Oldenburg Oldenburg Germany; ^4^ OnkoZert GmbH Neu‐Ulm Germany; ^5^ Federal Association of German Prostate Cancer Patient Support Groups Bonn Germany; ^6^ Kliniken Rhein‐Ruhr Herne Germany; ^7^ Martini‐Klinik Prostate Cancer Center Hamburg Hamburg Germany; ^8^ Klinikum Memmingen Memmingen Germany; ^9^ Caritas‐Krankenhaus Bad Mergentheim Germany; ^10^ University Hospital Erlangen Erlangen Germany; ^11^ KRH Klinikum Siloah Hannover Germany; ^12^ University Medical Center of Johannes Gutenberg University Mainz Mainz Germany; ^13^ University Hospital Ulm Ulm Germany

**Keywords:** health services research, multilevel analysis, prostate cancer, prostate neoplasms, psycho‐oncology, psychosocial oncology

## Abstract

**Background:**

Cancer patients often suffer from psychological distress. Psycho‐oncological services (POS) have been established in some health care systems in order to address such issues. This study aims to identify patient and center characteristics that elucidate the use of POS by patients in prostate cancer centers (PCCs).

**Methods:**

Center‐reported certification and patient survey data from 3094 patients in 44 certified PCCs in Germany were gathered in the observational study (Prostate Cancer Outcomes). A multilevel analysis was conducted.

**Results:**

Model 1 showed that utilization of POS in PCCs is associated with patients’ age (OR = 0.98; 95%‐CI = 0.96‐0.99; *P* < .001), number of comorbidities (1‐2 vs 0, OR = 1.27; 95%‐CI = 1.00‐1.60; *P*=.048), disease staging (localized high‐risk vs localized intermediate risk, OR = 1.41; 95%‐CI = 1.14‐1.74; *P* < .001), receiving androgen deprivation therapy before study inclusion (OR = 0.19; 95%‐CI = 0.10‐0.34; *P* < .001), and hospital teaching status (university vs academic, OR = 0.09; 95%‐CI = 0.02‐0.55; *P* = .009). Model 2 additionally includes information on treatment after study inclusion and shows that after inclusion, patients who receive primary radiotherapy (OR = 0.05; 95%‐CI = 0.03‐0.10; *P* < .001) or undergo active surveillance/watchful waiting (OR = 0.06; 95%‐CI = 0.02‐0.15; *P* < .001) are less likely to utilize POS than patients who undergo radical prostatectomy. Disease staging (localized high‐risk vs localized intermediate risk, OR = 1.31; 95%‐CI = 1.05‐1.62; *P* = .02) and teaching status (university vs academic, OR = 0.08; 95%‐CI = 0.01‐0.65; *P* = .02) are also significant predictors for POS use. The second model did not identify any other significant patient characteristics.

**Conclusions:**

Future research should explore the role of institutional teaching status and whether associations with therapy after study inclusion are due to treatment effects – for example, less need following radiotherapy – or because access to POS is more difficult for those receiving radiotherapy.

## Graphical abstract

The paper identifies patient and center characteristics that account for the use of psycho‐oncological services by prostate cancer patients in certified prostate cancer centers in Germany. Multilevel analysis was conducted. The results indicate that the varying utilization of psycho‐oncological services in certified prostate cancer centers may be better explained by characteristics of the centers and the patients’ clinical characteristics, rather than by the patients’ sociodemographic features.

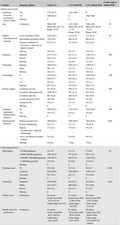

## 1 INTRODUCTION

Patients with cancer not only suffer from physical conditions, they also often have high levels of psychological distress.^1^ The psychological burden has been related to their reduced quality of life,^2^ as well as higher mortality in cancer patients.^3^ Among men, prostate cancer (PCa) is an important public health concern in many countries.^4^ The men affected often have to deal with impaired sexual function or incontinence^5^ and depression, or perceived stigma.^5^, ^6^ Psycho‐oncological services (POS) are an important and effective instrument for helping patients cope with cancer‐related mental illnesses.^7^

In view of the benefit that patients receive from POS, efforts have been made to establish psycho‐oncological services in various countries.^8^ In Germany, the German Cancer Society has set up a certification program to ensure high standards in cancer care, including the routine provision of POS for everyone in need.^9^ More than 100 prostate cancer centers (PCCs) currently hold certification in accordance with the German Cancer Society’s criteria, and they treat almost 50% of new prostate cancer cases in Germany.^10^ In order to receive certification, hospitals have to meet multiple requirements: among other things, certified centers are obliged to provide a multidisciplinary team including psychologists or physicians trained in psycho‐oncology. They also have to guarantee that every patient will receive psycho‐oncological screening using a valid instrument in accordance with the clinical guidelines,^11^ in order to assess the patients’ burden and need for support. In preparation for the certification audits, centers have to report the percentage of patients who actually utilize psycho‐oncological counseling for at least 25 minutes, either after positive screening or upon request. Rates of screening results do not have to be reported.

However, patients’ utilization of POS varies substantially among the different cancer centers in Germany.^9^, ^12^ Firstly, POS utilization varies between centers treating different entities. Utilization rates in PCCs are low in comparison with breast cancer centers, for example,^9^ which may be related to men expressing less need for psychological support than women.^13^ Secondly, differences in POS utilization across PCCs have been reported.^12^ Predisposing and enabling factors at the individual and contextual level, beyond need, have to be taken into consideration when explaining the use of health services.^14^ With regard to the utilization of POS in particular, it has been shown that centers that have held certification for a shorter period, as well as centers in rural areas and university hospitals, report significantly lower rates of POS utilization.^12^, ^15^ On the individual level, research indicates that patient characteristics such as age, educational level, and financial situation may influence psychological distress in cancer patients and their use of POS.^15^, ^16^, ^17^ In addition, the type of cancer therapy being received and disease progression may also have an effect on cancer patients’ psychological comorbidity and utilization of POS.^15^, ^16^, ^18^

The aim of the present analysis was to identify center‐related as well as sociodemographic and clinical patient characteristics that account for the utilization of POS by PCa patients. Two models were built: one model with individual patient and structural center characteristics as well as a second model additionally including information on process level. The identification of factors that impede or facilitate utilization is an important contribution to cancer‐related health care, as it may help ensure equal access to low‐threshold POS for all patients.

## 2 METHODS

### 2.1 Data collection

Data were collected in a prospective observational study Prostate Cancer Outcomes (PCO‐study), the German branch of the large‐scale cohort (TrueNTH Global Registry),^19^ in which providers and patients from several countries are taking part. The registry's aim is to improve the health of locally treated PCa patients by assessing and comparing clinical and patient‐reported outcomes. In Germany, centers certified by the German Cancer Society contribute to the registry by participating in the PCO‐study, which provides a uniform data collection infrastructure that has been described elsewhere (Kowalski et al, unpublished data, 2019). Eligibility criteria for patients were being diagnosed with a PCa for the first time (any T, any N, M0) and undergoing major parts of their treatment in one of the participating PCCs (local treatment as well as patients scheduled for active surveillance – AS – and watchful waiting – WW). In the PCO‐study, participating PCCs invite patients to give written informed consent for the collection of data. Patient‐reported outcome data and sociodemographic information are collected through a paper‐and‐pencil or online questionnaire^20^ and are linked to data collected routinely during the certification process – including disease and treatment information such as utilization of POS.

The ethics committee of the Berlin Medical Association approved the study protocol (Eth‐12/16). The current analysis used data collected between July 2016 and April 2018 in 44 certified PCCs throughout Germany.

### 2.2 Measures

Individual patient information on the utilization of POS, age, comorbidity, disease staging, and treatment was obtained from the center‐reported certification data. In this analysis, utilization of POS is a dichotomous measure (yes/no) and means receiving actual psychological counseling for at least 25 minutes, as reported in the certification system. POS utilization in this analysis does not provide any information either about the patients’ need for POS or about the screening results. The information was recorded while the patient was receiving treatment in the PCC. Age is a continuous measure (number of years); comorbidity is a number from the total of 12 predefined conditions listed in the protocol proposed by Martin et al^21^ (grouped into 0, 1, ≥ 2). Disease staging was calculated in accordance with the clinical guideline for the diagnosis and treatment of PCa (see Supporting Information I) – taking into account the prostate‐specific antigen value, Gleason score, and clinical stage categorized into five categories (localized low risk, localized intermediate risk, localized high risk, locally advanced, advanced). Treatment after inclusion in the study was grouped into four categories, comprising three local treatment types plus active surveillance and watchful waiting (radical prostatectomy, RPE; primary radiotherapy, primary RT; RPE + adjuvant RT; AS/WW), as these are the most common treatment options for patients with prostate cancer in Germany. Androgen deprivation therapy (ADT) before inclusion in the study was also noted. Information drawn from the patient survey included educational level, nationality, and insurance status (see Supporting Information II for questionnaire). Educational level was grouped into three categories (lower secondary school education, intermediate secondary school education, entrance certificate for university or university of applied sciences). Nationality was only grouped into German (including multiple nationalities) or other nationalities. Insurance status was grouped into the two categories of statutory and private. With regard to center characteristics, the analysis took into consideration information about the size of the municipality in which the center was located (population <20 000, 20 000‐100,000, >100 000‐1 000 000, >1 000 000), the number of primary cases (continuous), months since first certification (continuous), ownership (nonprofit, public, private), and teaching status (nonacademic, academic, university hospital) – all of which information is collected during the certification process. In Germany, university hospitals and academic hospitals both refer to institutions that train doctors. The difference is that academic hospitals have a contract with the medical faculty of a university, but do not belong to the university. In order to achieve interpretable odds ratios (OR), the number of primary cases was scaled (divided by 10).

### 2.3 Data analysis

Firstly, descriptive analyses were carried out for the dependent variable “POS utilization” and for the independent variables of the total sample, of the group of patient that received POS and the group of that did not utilize POS. Secondly, bivariate correlations were performed to identify statistically significant differences between patients who received POS and patients who did not receive POS (t‐test for continuous variables and Chi‐square test for categorial variables). Thirdly, multilevel modeling was performed for two levels: patients (level 1), nested in centers (level 2). A two‐level random intercept hierarchical logistic model without predictors (the null model) was calculated in order to determine the intraclass correlation coefficient (ICC). The ICC provides information about the proportion of variance that is attributable to differences between centers. Then, patient as well as center characteristics were added to the model in order to examine their relation to POS utilization. Sociodemographic information, information on disease staging and ADT prior to study inclusion are data on individual level and information on municipality, teaching status, ownership and primary cases are data on a structural level. However, information on treatment are – along with information on POS utilization – the only data on a process level in this analysis. In order to determine the best‐fitting model and to take possible mediating effects into account, information on treatment after inclusion in the study was added in a second model. For model comparison, the Akaike (AIC) and Bayesian (BIC) information criteria were also calculated. There were no missing data items for the independent metric variables. Missing cases in the independent categorical variables were included as separate categories in order to avoid case deletion. For the number of comorbidities, the default option in the documentation is zero, and patients are considered to have no comorbidities when no information is given. However, there were centers that did not modify the default value of zero for any of their patients, and it can be assumed that they did not report comorbidities at all. Patients from these centers were assigned a missing value for the number of comorbidities. In this context, a sensitivity analysis was carried out excluding all centers that did not report the number of comorbidities for any patient from the multilevel models. Another sensitivity analysis was conducted excluding all centers that included less than 30 patients in the study. Subsequent to the multilevel analysis, there was run an analysis of variance (ANOVA) was performed in order to analyze whether patients in the different therapy groups differ significantly in age. All statistical analyses were carried out using STATA, version 15.1 (StataCorp LLC, College Station, Texas, USA).

## 3 RESULTS

### 3.1 Participants and descriptive results

A total of 3094 patients in 44 PCCs took part in the PCO‐study, representing 39.3% of center patients who were eligible for inclusion (Kowalski et al, unpublished data, 2019). One thousand one hundred thirty‐four patients (36.7%) used POS for at least 25 minutes, while 1960 patients (63.4%) did not. Table 1 summarizes the descriptive results. Rates of POS utilization by study participants among the PCCs ranged from 0% to 100%, with a mean of 43.47% (SD 32.38) and a median of 34.4%.

**TABLE 1:** Descriptive results at the patient level for all patients in the sample (n = 3094), patients with psycho‐oncological services (POS, n = 1134) and without POS (n = 1960): frequencies, mean, standard deviation (SD), range; bivariate correlations to identify statistically significant differences between patients with POS and patients without POS (*P*‐values based on t‐test for continuous variable (age) and Chi‐square tests for categorical variables)

Variables	Response options	Total n (%)	n (%) with POS	n (%) without POS	*P*‐value with vs without POS
Patient characteristics					
Utilization of psycho‐oncological services (POS)	Yes	1134 (36.7)	1134 (100)	0	
	No	1960 (63.4)	0	1960 (100)	
	Missing	0	0	0	
Age	Continuous	3094 (100) Mean (SD): 66 (7.41) Range: 39‐85	1134 (100) Mean (SD): 65.87 (7.22) Range: 39‐84	1960 (100) Mean (SD): 66.05 (7.51) Range: 41‐85	.50
Highest educational level achieved	Lower secondary school	1186 (38.3)	474 (41.8)	712 (36.3)	.02
	Intermediate secondary school	730 (23.6)	264 (23.3)	466 (23.8)	
	Entrance certificate for university or university of applied sciences	975 (31.5)	331 (29.2)	644 (32.9)	
	Missing	203 (6.6)	65 (5.7)	138 (7.0)	
Insurance	Statutory	2275 (73.5)	877 (77.3)	1398 (71.3)	.002
	Private	662 (21.4)	212 (18.7)	450 (23.0)	
	Missing	157 (5.1)	45 (4.0)	112 (5.7)	
Nationality	German	2840 (91.8)	1051 (92.7)	1789 (91.3)	.75
	Other	109 (3.5)	42 (3.7)	67 (3.4)	
	Missing	145 (4.7)	41 (3.6)	104 (5.3)	
Comorbidity	0	1521 (49.2)	491 (43.3)	1030 (52.6)	.050
	1‐2	856 (27.7)	299 (26.4)	557 (28.4)	
	>2	50 (1.6)	25 (2.2)	25 (1.3)	
	Missing	667 (21.6)	319 (28.1)	348 (17.8)	
Disease staging	Localized, low risk	521 (16.8)	198 (17.5)	323 (16.5)	.005
	Localized, intermediate risk	1399 (45.2)	467 (41.2)	932 (47.6)	
	Localized, high risk	991 (32.0)	388 (34.2)	603 (30.8)	
	Locally advanced (T3/4)	144 (4.6)	66 (5.8)	78 (4.0)	
	Advanced (N1)	39 (1.3)	15 (1.3)	24 (1.2)	
	Missing	0	0	0	
Androgen deprivation therapy before inclusion	No	2992 (96.7)	1110 (97.9)	1882 (96.0)	.005
	Yes	102 (3.3)	24 (2.1)	78 (4.0)	
	Missing	0	0	0	
Treatment after inclusion	Radical prostatectomy	2608 (84.3)	1005 (88.6)	1603 (81.8)	<.001
	Primary radiotherapy	221 (7.1)	35 (3.1)	186 (9.5)	
	Radical prostatectomy + adjuvant radiotherapy	172 (5.6)	77 (6.8)	95 (4.9)	
	Active surveillance/watchful waiting	79 (2.6)	10 (0.9)	69 (3.5)	
	Missing	14 (0.5)	7 (0.6)	7 (0.4)	
Center characteristics					
Municipality	<20 000 population	32 (1.0)	15 (1.3)	17 (0.9)	.03
	20 000‐100 000 population	1067 (34.5)	417 (36.8)	650 (33.2)	
	>100 000‐1 000 000 population	1816 (58.7)	650 (57.3)	1166 (59.5)	
	>1 000 000 population	179 (5.8)	52 (4.6)	127 (6.5)	
	Missing	0	0	0	
Teaching status	No	123 (4.0)	43 (3.8)	80 (4.1)	<.001
	Academic	2474 (80.0)	1026 (90.5)	1448 (73.9)	
	University	497 (16.1)	65 (5.7)	432 (22.0)	
	Missing	0	0	0	
Ownership	Nonprofit	1576 (50.9)	632 (55.7)	944 (48.2)	<.001
	Public	1413 (45.7)	460 (40.6)	953 (48.6)	
	Private	105 (3.4)	42 (3.7)	63 (3.2)	
	Missing	0	0	0	
Primary cases	Continuous	44 centers Center mean (SD): 22.22 (32.18) Center range: 10.2‐225	41 centers Center mean (SD): 22.89 (33.26) Center range: 10.2‐225	42 centers Center mean (SD): 22.67 (32.89) Center range: 10.2‐225	
Months since first certification	Continuous	44 centers Center mean (SD): 80.21 (40.32) Center range: 0.43‐126.67	41 centers Center mean (SD): 80.44 (39.94) Center range: 0.43‐126.57	42 centers Center mean (SD): 80.48 (39.64) Center range: 0.43‐126.67 (42 centers)	

### 3.2 Multivariable results

The ICC for the null model shows that 50.3% of the variance in POS utilization is attributable to differences between the centers. Table 2 summarizes the multivariable results.

**TABLE 2:** Results of the logistic multilevel analyses: odds ratios (OR), *P*‐values and 95% confidence intervals (95% CI)

Variables	Response options	Model 1 Without treatment after study inclusion			Model 2 With treatment after study inclusion		
		OR	*P*‐value	95% CI	OR	*P*‐value	95% CI
Intercept		1.30	.77	0.21‐7.94	0.61	.64	0.08‐4.69
Patient characteristics							
Age	Continuous	**0.98**	**<.001**	**0.96‐0.99**	0.99	.11	0.98‐1.00
Highest educational level achieved	Lower secondary school	Reference group					
	Intermediate secondary school	0.86	.22	0.67‐1.09	0.85	.22	0.66‐1.10
	Entrance certificate for university or university of applied sciences	0.86	.22	0.68‐1.09	0.86	.23	0.68‐1.10
	Missing	1.05	.86	0.58‐1.91	1.06	.86	0.57‐1.98
Insurance	Statutory	Reference group					
	Private	1.00	.99	0.79‐1.27	0.97	.81	0.75‐1.24
	Missing	0.73	.50	0.29‐1.82	0.57	.28	0.21‐2.57
Nationality	German	Reference group					
	Other	1.03	.90	0.63‐1.70	0.92	.74	0.55‐1.54
	Missing	1.44	.51	0.49‐4.25	2.06	.24	0.62‐6.77
Comorbidity	0	Reference group					
	1‐2	**1.27**	**.048**	**1.00‐1.60**	1.12	.36	0.88‐1.43
	>2	1.65	.13	0.86‐3.17	1.43	.29	0.74‐2.76
	Missing	2.90	.08	0.87‐9.68	2.44	.21	0.61‐9.72
Disease staging	Localized, low risk	0.83	.18	0.64‐1.09	1.08	.58	0.82‐1.43
	Localized, intermediate risk	Reference group					
	Localized, high risk	**1.41**	**<.001**	**1.14‐1.74**	**1.31**	**.02**	**1.05‐1.62**
	Locally advanced (T3/4)	1.33	.18	0.87‐2.03	1.29	.26	0.83‐1.98
	Advanced (N1)	2.14	.08	0.91‐4.98	2.05	.12	0.83‐5.07
Androgen deprivation therapy before study inclusion	No	Reference group					
	Yes	**0.19**	**<.001**	**0.10‐0.34**	0.63	.15	0.34‐1.18
Treatment after study inclusion	Radical prostatectomy	Reference group					
	Primary radiotherapy				**0.05**	**<.001**	**0.03‐0.10**
	Radical prostatectomy + adjuvant radiotherapy				1.23	.32	0.81‐1.85
	Active surveillance/ watchful waiting				**0.06**	**<.001**	**0.02‐0.15**
	Missing				0.72	.62	0.20‐2.6
Center characteristics							
Municipality	< 20 000 population	0.79	.90	0.02‐28.17	0.49	.73	0.01‐29.71
	20 000‐100 000 population	0.86	.83	0.24‐3.17	1.08	.92	0.24‐4.84
	>100 000‐1 000 000 population	Reference group					
	>1 000 000 population	3.55	.36	0.24‐53.28	3.96	.39	0.18‐88.65
Teaching status	No	1.34	.76	0.20‐8.85	1.09	.94	0.13‐9.54
	Academic	Reference group					
	University	**0.09**	**.009**	**0.02‐0.55**	**0.08**	**.02**	**0.01‐0.65**
Ownership	Nonprofit	Reference group					
	Public	1.13	.85	0.32‐4.04	1.01	.98	0.24‐4.38
	Private	0.32	.39	0.02‐4.20	0.32	.46	0.02‐6.33
Primary cases	Continuous	1.00	.72	0.97‐1.02	0.99	.56	0.97‐1.02
Months since first certification	Continuous	1.01	.14	1.00‐1.03	1.02	.10	1.00‐1.03
Patients (n)		3094			3094		
Centers (n)		44			44		
Akaike‐Information‐Criterion		3144.45			2991.59		
Bayesian‐Information‐Criterion		3307.45			3178.74		
ICC (null model)		0.44 (0.50)			0.52 (0.50)		

Bold indicates significant results (*P* < .05).

*Model 1.* The first model indicated that the older the patients were, the less likely they were to use POS in the centers (OR 0.98; 95% CI, 0.96‐0.99). Comorbid patients with one or two additional conditions were significantly more likely to utilize POS than patients without any comorbidities (OR 1.27; 95% CI, 1.00‐1.60). Utilization of POS was significantly more likely in patients with localized high‐risk disease stages than in patients with localized intermediate‐risk stages (OR 1.41; 95% CI, 1.14‐1.74). Patients who had received ADT (OR 0.19; 95% CI, 0.10‐0.34) before inclusion in the study were significantly less likely to use POS than patients who had not. With regard to the center characteristics, model 1 shows that patients in university hospitals were significantly less likely to utilize POS than those in other academic teaching hospitals (OR 0.09; 95% CI, 0.02‐0.55). For model 1, the AIC was 3144.45 and the BIC 3307.45. These values need to be interpreted in the context of the second model (see below).

*Model 2.* With additional adjustment for treatment after inclusion in the study, patients with localized high‐risk disease stages were still found to be more likely to use POS than patients with localized intermediate‐risk stages (OR 1.31, 95% CI, 1.05‐1.62). University hospitals were also still associated with lower POS utilization than academic teaching hospitals (OR 0.08; 95% CI, 0.01‐0.65). However, age (OR 0.99; 95% CI, 0.98‐1.00), comorbidity (OR 1.12; 95% Cl, 0.88‐1.43), and having received ADT before inclusion in the study (OR 0.63; 95% CI, 0.34‐1.18) were not confirmed as significant predictors for POS utilization after adjustment for treatment after study inclusion. This model also showed that patients who received primary RT (OR 0.05; 95% CI, 0.03‐0.10), as well as patients who underwent AS or WW (OR 0.06; 95% CI, 0.02‐0.15), were significantly less likely to use POS than patients who received RPE. For the second model, the AIC was 2991.59 and the BIC 3178.74 – indicating better model adjustment than model 1.

## 4 DISCUSSION

This analysis indicates that varying rates of POS utilization in German prostate cancer centers may be better explained by the organizational characteristics of the centers concerned and by the patients’ clinical characteristics, rather than by the patients’ sociodemographic features. Two multilevel models were built in the analysis – one model taking account of patient and hospital characteristics, but without adjustment for treatment after inclusion in the study (model 1); and the other additionally taking account of treatment variables after inclusion in the study (model 2). The measurements for goodness of fit (AIC and BIC) that were investigated indicate overall that the model taking account of treatment after inclusion in the study explains POS utilization better than the first model.

The fully adjusted model 2 indicates the following associations: Firstly, it shows that POS is less likely to be used in university hospitals than in other academic hospitals, even with adjustment for patient characteristics. This corresponds to the findings reported by Kowalski et al,^12^ who observed lower rates of POS utilization in university hospitals in comparison with cancer centers in general. However, there is a lack of information about how to interpret this finding, since the data in the analysis provide information about actual utilization of POS, but do not give any information regarding whether or not the services used meet the patients’ needs (centers’ screening results describing patients’ psycho‐oncological needs are not available, and it has not been documented whether patients ask for POS despite negative screening results). The mean utilization rate for POS in university hospitals in the present sample was 14%, which is below the prevalence rate for mental disorders in prostate cancer patients, according to Mehnert et al^1^ POS staff‐patient ratio is predefined by the certification requirements for all prostate cancer centers, thus, it should not differ between centers with different teaching status. However, Ansmann et al^22^ found that, in comparison with employees in non‐teaching hospitals, more employees in university hospitals consider that there are problems with collaboration among special areas of responsibility in hospital departments. However, psycho‐oncological care requires interaction among different units – for example, in order to communicate screening results. Process problems or internal departmental boundary problems may therefore represent barriers in university hospitals to successful utilization of POS. The size of the effect needs to be interpreted with caution, since the sample included patients from only seven university hospitals, which may not be representative.

Secondly, model 2 indicates that patients who receive RPE or RPE plus adjuvant radiotherapy are more likely to utilize POS than AS/WW patients or those receiving primary radiotherapy after inclusion in the study. This might be related to the often‐mentioned adverse effects of RPE, which in comparison with radiotherapy and active monitoring can severely impede sexual function and urinary continence.^23^, ^24^ This may also indicate differences between the in‐patient and outpatient settings and may suggest deficiencies in the organization of care^25^ – that is, patients who receive RPE may have better access to POS than patients with primary radiotherapy or those who are undergoing AS/WW. Further research is needed here.

Thirdly, model 2 shows a trend suggesting that patients with localized high‐risk cancer, patients with locally advanced cancer (T3/4), and patients with advanced cancer (N1) use POS more often than patients who have localized cancer and an intermediate risk level. However, significance was only reached in this regard for localized high‐risk cancer in comparison with localized intermediate‐risk cancer, probably due to the small number of cases of locally advanced and advanced cancer. These findings are consistent with previous research.^15^, ^16^, ^17^

Model 1, which does not include treatment after inclusion in the study, shows the same trends for the hospitals’ teaching status and patients’ disease staging as model 2. Moreover, it also demonstrates significant effects for age. The association between age and POS utilization does not persist after adjustment for treatment types (model 2). An additional analysis (Supporting Information III Table 3.1) showed that patients who received primary radiotherapy and AS or WW were significantly older than those with RPE or RPE + adjuvant radiotherapy. This indicates a spurious correlation between age and POS utilization. Moreover, in model 1 in the present analysis, patients who received ADT before inclusion in the study made significantly less use of POS than patients who did not receive such treatment before the survey. These effects disappear after controlling for treatment after inclusion in the study. The negative association between ADT and POS utilization is not consistent with previous research indicating that the tremendous physical and hormonal changes involved entail high levels of psychological distress.^18^ Possible explanations for these inconsistent results might be that these patients had already had an opportunity to cope with the distressing consequences of the disease, or that they had already received POS in an outpatient setting. In addition, the significant effect of comorbidity in the first model becomes marginal after adjustment for therapeutic details in the second model. One explanation for this might be that treatment decisions are related to the patients’ comorbid conditions.

The most important limitation of the present study is that the numbers of patients with primary radiotherapy and AS/WW were low in comparison with RPE patients. The particular high values for treatment after study inclusion could be due to selection effects and sample distribution. It therefore needs to be considered whether the patients with primary radiotherapy and AS/WW who were included were representative of the population, or whether they were in above‐average condition and thus may have led to bias in the results. The numbers of AS/WW patients may be low because they are often treated as outpatients by office‐based physicians. The patients included in the present study were relatively young,^26^ slightly better educated, and with an above‐average rate of private insurance coverage in comparison with the overall population of patients with prostate cancer in Germany, and this needs to be taken into consideration before the results are generalized (Kowalski et al, unpublished data, 2019). In addition, the analysis includes a large number of missing values for numbers of comorbidities, partly due to the way in which comorbidity data are collected, as mentioned above (Kowalski et al, unpublished data, 2019).^27^ Since the default value in the documentation system is zero, patients are considered to have no comorbidities if no information is given. For centers that did not modify the default value of zero for any of their patients, the number of comorbidities was therefore treated as a missing value for all of the patients from those centers in the analysis. In addition, a sensitivity analysis (Supporting Information III Table 3.2 and 2.3) was carried out by excluding from the multilevel models all centers that did not report the number of comorbidities for any patient, and this did not show any results strongly diverging from the original models. However, this issue involving comorbidity data needs to be taken into consideration when interpreting the results of the analysis. The numbers of patients included in the study also varied widely between the different centers, ranging from four to 431 patients. A small number of recruited patients in some of the centers, along with the fact that the dependent variable is binary, mean that some centers never, or very rarely, satisfy one of the binary variable categories. This may explain the very high ICC of 50.3% in the null model in this analysis, which should therefore be interpreted with caution. An additional sensitivity analysis without centers that included fewer than 30 patients showed the same effect trends as the original models that included all of the centers (Supporting Information III Table 3.4 and 3.5). The high ICC in the second model also indicates that much of the variation between hospitals remains unexplained. It should also be noted that the PCCs that opted for inclusion in this sample may be more dedicated than other PCCs in Germany generally that were not participating in the PCO‐study, and this might bias the results. On a positive note, the information about POS utilization was drawn from centers’ records rather than from patient surveys, and the exact numbers of patients in centers were known, so that any selection among study participants could be analyzed. In addition, the multilevel data at the structural, process, and individual levels represent a strength of the analysis, as they allowed adjustment for center characteristics as well as for patient characteristics and did not restrict the analysis to a single level. Another strength of the analysis is the large sample size.

The following implications for future research can be drawn from the results of this analysis. Firstly, further research is needed in order to determine whether the differences in POS utilization are due to treatment effects or due to patients receiving primary radiotherapy or undergoing AS/WW who are underrepresented in the present sample. If the latter proves to be the case, then ways of including more radiotherapy and AS/WW patients need to be developed. In general, the inclusion difficulties described also emphasize the relevance of increasing the use of quality‐assurance data in health care research, which could counteract inclusion issues related to study data. If treatment effects were instead to be confirmed as causing differences in POS utilization, the reasons behind this should be investigated. Secondly, future qualitive research needs to find an explanation for the situation that psycho‐oncology services are less likely to be used in university hospitals than in other academic teaching hospitals. It needs to be investigated whether this is due to a discrepancy between the patients’ need for psycho‐oncological support and the provision of access to POS, in order to identify potential problems and develop measurements that address structural difficulties in psycho‐oncological health care. Above, screening results should be documented in the routine in addition to POS utilization in order to identify to what extent utilization patterns of POS are according to an objectifiable need. The extent to which screening methods for psycho‐oncological need vary across centers should be examined, as well as the way in which this influences the process – for example, how it can be ensured that patients who are screened as negative for psycho‐oncological need may still receive POS if they wish to. Further potential confounder variables like partnership status and psychological comorbidity should be considered in future research, since literature indicates correlations with utilization of POS but insufficient data was available for this analysis.^1^, ^16^, ^25^

## CONFLICT OF INTEREST

CB, CK, SD, AO, and SW are employees of the two institutions in charge of the certification system. RR, LA, EGC, GF, PB, BB, RB, JE, FK, SJO, IT, and FZ hereby declare that they have no potential conflicts of interest.

## AUTHORS’ CONTRIBUTIONS

Clara Breidenbach: formal analysis, writing – original draft. Rebecca Roth: writing – review and editing. Lena Ansmann: writing – review and editing. Simone Wesselmann: conceptualization, writing – review and editing. Sebastian Dieng: conceptualization, data curation, writing – review and editing. Ernst‐Günther Carl: conceptualization, writing – review and editing. Günter Feick: conceptualization, writing – review and editing. Alisa Oesterle: data curation, writing – review and editing. Peter Bach: investigation, writing – review and editing. Burkhard Beyer: investigation, writing – review and editing. Rainer Borowitz: investigation, writing – review and editing. Jörg Erdmann: investigation, writing – review and editing. Frank Kunath: investigation, writing – review and editing. Simba‐Joshua Oostdam: investigation, writing – review and editing. gor Tsaur: investigation, writing – review and editing. riedemann Zengerling: investigation, writing – review and editing. Christoph Kowalski: conceptualization, methodology, project administration, formal analysis, writing – original draft.

## Supporting information

Supplementary Material

Supplementary Material

Supplementary Material
