# Supplementary material for: Use of psycho‐oncological services by prostate cancer patients: A multilevel analysis
Source: Cancer Med. 2020 Mar 31;9(11):3680–90. doi: 10.1002/cam4.2999 (PMC7286449; doi:10.1002/cam4.2999)
Supplement: Supplementary file 1 — Supplementary Material [file CAM4-9-3680-s001.docx]

**Supporting Information I**

**Staging according to the German Guideline Prostate Cancer (version May 2019)**

In this study, we use the staging proposed by the German Guideline for Prostate Cancer^1^.

- Localized prostate cancer: T1-2, N0, M0
  - Localized prostate cancer with low risk: PSA ≤ 10ng/ml and Gleason-Score 6 and cT1c or cT2a
  - Localized prostate cancer with intermediate risk: PSA >10ng/ml – 30ng/ml or Gleason 7 or cT 2b
  - Localized prostate cancer with high risk: PSA > 20ng/ml or Gleason ≥ 8 or cT2c
- Locally advanced prostate cancer: T3-4, N0, M0
- Advanced/metastasized prostate cancer: any T N1 and/or M1

Furthermore, localized prostate cancer with cT1a or cT1b is classified as localized prostate cancer with low risk.

1. Leitlinienprogramm Onkologie (Arbeitsgemeinschaft der Wissenschaftlichen Medizinischen Fachgesellschaften e. V., Deutsche Krebsgesellschaft e. V., Deutsche Krebshilfe e. V.). Interdisziplinäre Leitlinie der Qualität S3 zur Früherkennung, Diagnose und Therapie der ver-schiedenen Stadien des Prostatakarzinoms. Berlin: Leitlinienprogramm Onkologie; Published May 2019. <https://www.leitlinienprogramm-onkologie.de/leitlinien/prostatakarzinom/>. Accessed December, 12 2019.
