# Supplementary material for: Use of psycho‐oncological services by prostate cancer patients: A multilevel analysis
Source: Cancer Med. 2020 Mar 31;9(11):3680–90. doi: 10.1002/cam4.2999 (PMC7286449; doi:10.1002/cam4.2999)
Supplement: Supplementary file 2 — Supplementary Material [file CAM4-9-3680-s002.pdf]

label username

Please fill out today's date

Day		Month		Year			
☐	☐	☐	☐	☐	☐	☐	☐

Please chose the best fitting response for your situation in the last weeks. It is important that you answer all the questions.

1. Over the past 4 weeks, how often have you leaked urine?

- ☐ More than once a day
- ☐ About once a day
- ☐ More than once a week
- ☐ About once a week
- ☐ Rarely or never

(select one answer)

2. Which of the following best describes your urinary control during the last 4 weeks?

- ☐ No urinary control whatsoever
- ☐ Frequent dribbling
- ☐ Occasional dribbling
- ☐ Total control

(select one answer)

3. How many pads or adult diapers per day did you usually use to control leakage during the last 4 weeks?

- ☐ None
- ☐ 1 pad per day
- ☐ 2 pads per day
- ☐ 3 or more pads per day

(select one answer)

4. How big a problem, if any, has each of the following been for you during the last 4 weeks?

(select one answer for each question)

	No problem	Very small problem	Small problem	Moderate problem	Big problem
a) Dripping or leaking urine	☐	☐	☐	☐	☐
b) Pain or burning on urination	☐	☐	☐	☐	☐
c) Bleeding with urination	☐	☐	☐	☐	☐
d) Weak urine stream or in-complete emptying	☐	☐	☐	☐	☐
e) Need to urinate frequently during the day	☐	☐	☐	☐	☐

5. Overall, how big a problem has your urinary function been for you during the last 4 weeks?

(select one answer)

- ☐ No problem
- ☐ Very small problem
- ☐ Small problem
- ☐ Moderate problem
- ☐ Big problem

6. How big a problem, if any, has each of the following been for you?

(select one answer for each question)

	No problem	Very small problem	Small problem	Moderate problem	Big problem
a) Urgency to have a bowel movement	☐	☐	☐	☐	☐
b) Increased frequency of bowel movements	☐	☐	☐	☐	☐
c) Losing control of your stools	☐	☐	☐	☐	☐
d) Bloody stools	☐	☐	☐	☐	☐
e) Abdominal/ Pelvic/Rectal pain	☐	☐	☐	☐	☐

7. Overall, how big a problem have your bowel habits been for you during the last 4 weeks?

(select one answer)

- ☐ No problem
- ☐ Very small problem
- ☐ Small problem
- ☐ Moderate problem
- ☐ Big problem

8. How would you rate each of the following during the last 4 weeks?

(select one answer for each question)

	Very poor to none	Poor	Fair	Good	Very good
a) Your ability to have an erection?	☐	☐	☐	☐	☐
b) Your ability to reach orgasm (climax)?	☐	☐	☐	☐	☐

9. How would you describe the usual QUALITY of your erections during the last 4 weeks?

(select one answer)

- ☐ None at all
- ☐ Not firm enough for any sexual activity
- ☐ Firm enough for masturbation and foreplay only
- ☐ Firm enough for intercourse

10. How would you describe the FREQUENCY of your erections during the last 4 weeks?

- ☐ I NEVER had an erection when I wanted one
- ☐ I had an erection LESS THAN HALF the time I wanted one
- ☐ I had an erection ABOUT HALF the time I wanted one
- ☐ I had an erection MORE THAN HALF the time I wanted one
- ☐ I had an erection WHENEVER I wanted one

(select one answer)

11. Overall, how would you rate your ability to function sexually during the last 4 weeks?

- ☐ Very poor
- ☐ Poor
- ☐ Fair
- ☐ Good
- ☐ Very good

(select one answer)

12. Overall, how big a problem has your sexual function or lack of sexual function been for you during the last 4 weeks?

- ☐ No problem
- ☐ Very small problem
- ☐ Small problem
- ☐ Moderate problem
- ☐ Big problem

(select one answer)

13. How big a problem during the last 4 weeks, if any, has each of the following been for you?

(select one answer for each question)

	No problem	Very small problem	Small problem	Moderate problem	Big problem
a) Hot flashes	☐	☐	☐	☐	☐
b) Breast tender-ness/enlargement	☐	☐	☐	☐	☐
c) Feeling depressed	☐	☐	☐	☐	☐
d) Lack of energy	☐	☐	☐	☐	☐
e) Change in body weight	☐	☐	☐	☐	☐

14. During the last 4 weeks, to what extent were you interested in sex?

- ☐ Not at all
- ☐ A little
- ☐ Quite a bit
- ☐ Very much

(select one answer)

15. Have you used any medications or devices to aid or improve erections?

☐ No

☐ Yes

(select one answer)

16. For each of the following medicines or devices, please indicate whether or not you have tried it or currently use it to improve your erections?

(select one answer for each question)

	Have not tried it	Tried it but was not helpful	It helped but I am not using it now	It helped and I use it sometimes	It helped and I always use it
a) Viagra or other pill	☐	☐	☐	☐	☐
Name of the pill					
b) **Muse** (intra-urethral alprostadil suppository)	☐	☐	☐	☐	☐
c) Penile injection therapy	☐	☐	☐	☐	☐
d) Vacuum erection device	☐	☐	☐	☐	☐
e) Other (name medica-tion/device if not listed)	☐	☐	☐	☐	☐
Name of the medication/device					

17. What citizenship do you hold?

☐ German (and possibly additional citizenship(s))

☐ Other

(select one answer)

18. Of the following, what is closest to your health insurance coverage?

☐ Statutory health insurance

☐ Private health insurance

☐ Other / none

(select one answer)

19. Of the following, what is the highest level of education you have successfully completed (usually by obtaining a certificate or diploma)?

☐ Lower secondary school or equivalent (8/9 years of schooling)

☐ Intermediate secondary school (10 years of schooling)

☐ Comprehensive school

☐ Entrance certificate for a higher technical college/university of applied science

☐ University entrance certificate

☐ Other

☐ None

(select one answer)

Thank you for taking part in the survey!
In this way you contribute to improving the treatment of patients with prostate cancer.
Please use the enclosed **return envelope** to give the questionnaire back where it was given to you or place it in the nearest letterbox (postage paid by the addressee).
