# Supplementary material for: Use of psycho‐oncological services by prostate cancer patients: A multilevel analysis
Source: Cancer Med. 2020 Mar 31;9(11):3680–90. doi: 10.1002/cam4.2999 (PMC7286449; doi:10.1002/cam4.2999)
Supplement: Supplementary file 3 — Supplementary Material [file CAM4-9-3680-s003.docx]

Supporting Information III – Further analyses

**TABLE 3.1
Results of an ANOVA comparing age mean per therapy type**

| **Therapy type** | **F** | **Age** | | |
| --- | --- | --- | --- | --- |
|  |  | **Mean** | **Standard Deviation** | **Frequency** |
| Radical prostatectomy | <.001 | 65.37^a^ | 7.25 | 2,608 |
| Primary radiotherapy |  | 70.99^b^ | 6.96 | 221 |
| Radical prostatectomy + adjuvant radiotherapy |  | 66.80^a^ | 6.66 | 172 |
| Active surveillance/ watchful waiting |  | 70.96^b^ | 7.72 | 79 |

^a,b^ groups with different code letters differ significantly (*P* < .001)

**TABLE 3.2
Results of the logistic multilevel analyses excluding all centers that did not report the number of comorbidities for any patient: odds ratios (OR), p-values and 95% confidence intervals (95% CI).**

| **Variables** | **Response options** | **Model 1  Without treatment after study inclusion** | | | **Model 2  With treatment after study inclusion** | | |
| --- | --- | --- | --- | --- | --- | --- | --- |
|  |  | OR | p-value | 95% CI | OR | p-value | 95% CI |
| Intercept |  | 2.10 | .56 | 0.18-25.11 | 0.87 | .92 | 0.05-15.18 |
| **Patient characteristics** | | | | | | | |
| Age | Continuous | **0.97** | **<.001** | **0.96-0.99** | 0.99 | .08 | 0.97-1.00 |
| Highest educational level achieved | Lower secondary school | Reference group | | | | | |
|  | Intermediate secondary school | 0.78 | .09 | 0.59-1.03 | 0.80 | .13 | 0.6-1.07 |
|  | Entrance certificate for university or university of applied sciences | 0.84 | .20 | 0.65-1.09 | 0.85 | .23 | 0.65-1.11 |
|  | Missing | 1.02 | .96 | 0.53-1.96 | 1.10 | .80 | 0.55-2.17 |
| Insurance | Statutory | Reference group | | | | | |
|  | Private | 1.09 | .52 | 0.84-1.42 | 1.05 | .72 | 0.80-1.38 |
|  | Missing | 0.66 | .49 | 0.21-2.10 | 0.55 | .36 | 0.15-1.99 |
| Nationality | German | Reference group | | | | | |
|  | Other | 1.01 | .96 | 0.58-1.77 | 0.93 | .80 | 0.53-1.64 |
|  | Missing | 1.82 | .36 | 0.50-6.61 | 2.30 | .25 | 0.55-9.63 |
| Comorbidity | 0 | Reference group | | | | | |
|  | 1–2 | **1.28** | **.04** | **1.01-1.61** | 1.14 | .30 | 0.89-1.45 |
|  | >2 | 1.67 | .13 | 0.87-3.21 | 1.44 | .28 | 0.74-2.78 |
| Disease staging | Localized, low risk | 0.89 | .45 | 0.66-1.21 | 1.09 | .60 | 0.79-1.50 |
|  | Localized, intermediate risk | Reference group | | | | | |
|  | Localized, high risk | **1.45** | **.002** | **1.15-1.82** | **1.34** | **.02** | **1.05-1.70** |
|  | Locally advanced (T3/4) | 1.51 | .08 | 0.95-2.38 | 1.42 | .15 | 0.89-2.27 |
|  | Advanced (N1) | 2.04 | .19 | 0.71-5.87 | 1.98 | .22 | 0.66-5.97 |
| Androgen deprivation therapy before study inclusion | No | Reference group | | | | | |
|  | Yes | **0.18** | **<.001** | **0.10-0.35** | 0.62 | .16 | 0.32-1.21 |
| Treatment after study inclusion | Radical prostatectomy | Reference group | | | | | |
|  | Primary radiotherapy |  | | | **0.07** | **<.001** | **0.04-0.14** |
|  | Radical prostatectomy + adjuvant radiotherapy |  |  |  | 1.07 | .78 | 0.67-1.72 |
|  | Active surveillance/ watchful waiting |  |  |  | **0.11** | **<.001** | **0.03-0.35** |
|  | Missing |  |  |  | 0.75 | .66 | 0.21-2.67 |
| **Center characteristics** | | | | | | | |
| Municipality | < 20,000 population | 2.27 | .68 | 0.05-108.64 | 1.22 | .93 | 0.01-112.39 |
|  | 20,000–100,000 population | 3.12 | .20 | 0.55-17.60 | 3.15 | .27 | 0.42-23.84 |
|  | > 100,000–1,000,000 population | Reference group | | | | | |
|  | > 1,000,000 population | 3.19 | .45 | 0.16-63.03 | 3.89 | .44 | 0.12-125.91 |
| Teaching status | No | 1.26 | .86 | 0.09-17.01 | 1.36 | .84 | 0.07-28.37 |
|  | Academic | Reference group | | | | | |
|  | University | 0.09 | .07 | 0.01-1.18 | 0.09 | .11 | 0.00-1.73 |
| Ownership | Nonprofit | Reference group | | | | | |
|  | Public | 0.71 | .71 | 0.12-4.16 | 0.72 | .76 | 0.09-5.70 |
| Primary cases | Continuous | 1.00 | .96 | 0.96-1.03 | 1.00 | .80 | 0.97-1.03 |
| Months since first certification | Continuous | 1.00 | .80 | 0.98-1.03 | 1.01 | .55 | 0.98-1.04 |
| Patients (n) |  | 2,427 | | | 2,427 | | |
| Centers (n) |  | 29 | | | 29 | | |
| Akaike-Information-Criterion |  | 2502.25 | | | 2409.604 | | |
| Bayesian-Information-Criterion |  | 2647.111 | | | 2577.642 | | |
| ICC (null  model) |  | 0.46 (0.54) | | | 0.54 (0.54) | | |

**TABLE 3.3
Descriptive results at the patient level (n=2,427) excluding all centers that did not report the number of comorbidities for any patient**

| **Variables** | **Response options** | **Total n (%)** |
| --- | --- | --- |
| **Patient characteristics** | | |
| Utilization of psycho-oncological services (POS) | Yes | 815 (33.6) |
|  | No | 1,612 (66.4) |
|  | Missing | 0 |
| Age | Continuous | 2,427 (100)  Mean (SD): 66.00 (7.47)  Range: 39-85 |
| Highest educational level achieved | Lower secondary school | 902 (37.2) |
|  | Intermediate secondary school | 541 (22.3) |
|  | Entrance certificate for university or university of applied sciences | 797 (32.8) |
|  | Missing | 187 (7.7) |
| Insurance | Statutory | 1,743 (71.8) |
|  | Private | 540 (22.3) |
|  | Missing | 144 (5.9) |
| Nationality | German | 2,206 (90.9) |
|  | Other | 84 (3.5) |
|  | Missing | 137 (5.6) |
| Comorbidity | 0 | 1,521 (62.7) |
|  | 1–2 | 856 (35.3) |
|  | > 2 | 50 (2.1) |
|  | Missing | 0 |
| Disease staging | Localized, low risk | 357 (14.7) |
|  | Localized, intermediate risk | 1,131 (46.6) |
|  | Localized, high risk | 796 (32.8) |
|  | Locally advanced (T3/4) | 117 (4.8) |
|  | Advanced (N1) | 26 (1.1) |
|  | Missing | 0 |
| Androgen deprivation therapy before inclusion | No | 2,334 (96.2) |
|  | Yes | 93 (3.8) |
|  | Missing | 0 |
| Treatment after inclusion | Radical prostatectomy | 2,050 (84.5) |
|  | Primary radiotherapy | 197 (8.1) |
|  | Radical prostatectomy + adjuvant radiotherapy | 120 (4.9) |
|  | Active surveillance/ watchful waiting | 46 (1.9) |
|  | Missing | 14 (0.6) |
| **Center characteristics** | | |
| Municipality | < 20,000 population | 32 (1.3) |
|  | 20,000–100,000 population | 744 (30.7) |
|  | > 100,000–1,000,000 population | 1,472 (60.7) |
|  | > 1,000,000 population | 179 (7.4) |
|  | Missing | 0 |
| Teaching status | No | 106 (4.4) |
|  | Academic | 1,868 (77.0) |
|  | University | 453 (18.7) |
|  | Missing | 0 |
| Ownership | Nonprofit | 1,185 (48.8) |
|  | Public | 1,242 (51.2) |
|  | Private | 0 |
|  | Missing | 0 |
| Primary cases | Continuous | 29 centers  Center mean (SD): 22.82 (33.28) Center range: 10.2–225 |
| Months since first certification | Continuous | 29 centers  Center mean (SD): 85.89 (36.21) Center range: 7.6–126.67 |

**TABLE 3.4**
**Results of the logistic multilevel analyses excluding all centers that included less than 30 patients in the study: odds ratios (OR), p-values and 95% confidence intervals (95% CI).**

| **Variables** | **Response options** | **Model 1  Without treatment after study inclusion** | | | **Model 2  With treatment after study inclusion** | | |
| --- | --- | --- | --- | --- | --- | --- | --- |
|  |  | OR | p-value | 95% CI | OR | p-value | 95% CI |
| Intercept |  | 0.85 | 0.888 | 0.09-7-90 | 0.34 | 0.383 | 0.03-3.79 |
| **Patient characteristics** | | | | | | | |
| Age | Continuous | **0.97** | **<.001** | **0.96-0.99** | 0.99 | .07 | 0.97-1.00 |
| Highest educational level achieved | Lower secondary school | Reference group | | | | | |
|  | Intermediate secondary school | 0.84 | .19 | 0.65-1.09 | 0.84 | .21 | 0.65-1.10 |
|  | Entrance certificate for university or university of applied sciences | 0.83 | .14 | 0.65-1.06 | 0.83 | .15 | 0.65-1.10 |
|  | Missing | 1.12 | .71 | 0.61-2.08 | 1.14 | .68 | 0.60-2.17 |
| Insurance | Statutory | Reference group | | | | | |
|  | Private | 1.03 | .79 | 0.81-1.33 | 1.00 | .99 | 0.78-1.30 |
|  | Missing | 0.68 | .45 | 0.25-1.85 | 0.59 | .34 | 0.20-1.74 |
| Nationality | German | Reference group | | | | | |
|  | Other | 1.01 | .96 | 0.60-1.71 | 0.93 | .79 | 0.54-1.59 |
|  | Missing | 1.32 | .63 | 0.42-4.10 | 1.76 | .37 | 0.51-6.04 |
| Comorbidity | 0 | Reference group | | | | | |
|  | 1–2 | 1.23 | .09 | 0.97-1.57 | 1.07 | .58 | 0.84-1.37 |
|  | >2 | 1.52 | .21 | 0.79-2.94 | 1.29 | .45 | 0.66-2.52 |
|  | Missing | 1.93 | .44 | 0.36-10.39 | 1.56 | .63 | 0.25-9.70 |
| Disease staging | Localized, low risk | 0.88 | .36 | 0.67-1.16 | 1.11 | .49 | 0.90-2.18 |
|  | Localized, intermediate risk | Reference group | | | | | |
|  | Localized, high risk | **1.38** | **.004** | **1.11-1.72** | **1.28** | **.03** | **1.02-1.60** |
|  | Locally advanced (T3/4) | 1.44 | .10 | 0.94-2.22 | 1.40 | .14 | 0.90-2.18 |
|  | Advanced (N1) | 2.05 | .13 | 0.81-5.17 | 1.97 | .18 | 0.74-5.25 |
| Androgen deprivation therapy before study inclusion | No | Reference group | | | | | |
|  | Yes | **0.17** | **<.001** | **0.09-0.33** | 0.57 | .09 | 0.29-1.10 |
| Treatment after study inclusion | Radical prostatectomy | Reference group | | | | | |
|  | Primary radiotherapy |  | | | **0.06** | **<.001** | **0.03-0.12** |
|  | Radical prostatectomy + adjuvant radiotherapy |  |  |  | 1.28 | .24 | 0.85-1.95 |
|  | Active surveillance/ watchful waiting |  |  |  | **0.08** | **<.001** | **0.03-0.23** |
|  | Missing |  |  |  | 0.87 | .84 | 0.22-3.49 |
| **Center characteristics** | | | | | | | |
| Municipality | < 20,000 population | 1.03 | .99 | 0.02-44.14 | 0.57 | .79 | 0.01-33.37 |
|  | 20,000–100,000 population | 1.89 | .71 | 0.25-7.75 | 1.49 | .68 | 0.23-9.69 |
|  | > 100,000–1,000,000 population | Reference group | | | | | |
|  | > 1,000,000 population | 6.63 | .30 | 0.19-228.27 | 7.41 | .31 | 0.16-344.93 |
| Teaching status | No | 0.89 | .93 | 0.06-12.88 | 0.86 | .92 | 0.05-15.59 |
|  | Academic | Reference group | | | | | |
|  | University | 0.19 | .15 | 0.02-1.77 | 0.17 | .15 | 0.02-1.87 |
| Ownership | Nonprofit | Reference group | | | | | |
|  | Public | 1.24 | .82 | 0.20-7.66 | 1.49 | .69 | 0.20-10.81 |
|  | Private | 0.56 | .66 | 0.04-7.88 | 0.73 | .83 | 0.04-13.01 |
| Primary cases | Continuous | 1.00 | .58 | 1.00-1.00 | 1.00 | -46 | 1.00-1.00 |
| Months since first certification | Continuous | 1.00 | .20 | 1.00-1.00 | 1.00 | .13 | 1.00-1.00 |
| Patients (n) |  | 2,833 | | | 2,833 | | |
| Centers (n) |  | 28 | | | 28 | | |
| Akaike-Information-Criterion |  | 2876.908 | | | 2758.981 | | |
| Bayesian-Information-Criterion |  | 3037.533 | | | 2943.403 | | |
| ICC (null  model) |  | 0.42 (0.46) | | | 0.46 (0.46) | | |

**TABLE 3.5**
**Descriptive results at the patient level (n = 2,833) excluding all centers that including less than 30 patients in the study: frequencies, mean, standard deviation (SD), range**

| **Variables** | **Response options** | **Total n (%)** |
| --- | --- | --- |
| **Patient characteristics** | | |
| Utilization of psycho-oncological services (POS) | Yes | 1,004 (35.4) |
|  | No | 1,829 (64.6) |
|  | Missing | 0 |
| Age | Continuous | 2,833 (100)  Mean (SD): 66.01 (7.38)  Range: 39-85 |
| Highest educational level achieved | Lower secondary school | 1,080 (38.1) |
|  | Intermediate secondary school | 654 (23.1) |
|  | Entrance certificate for university or university of applied sciences | 904 (31.9) |
|  | Missing | 195 (6.9) |
| Insurance | Statutory | 2,065 (72.9) |
|  | Private | 616 (21.7) |
|  | Missing | 152 (5.4) |
| Nationality | German | 2,591 (91.5) |
|  | Other | 100 (3.5) |
|  | Missing | 142 (5.0) |
| Comorbidity | 0 | 1,444 (51.0) |
|  | 1–2 | 800 (28.2) |
|  | > 2 | 49 (1.7) |
|  | Missing | 540 (19.1) |
| Disease staging | Localized, low risk | 472 (16.7) |
|  | Localized, intermediate risk | 1,291 (45.6) |
|  | Localized, high risk | 905 (31.9) |
|  | Locally advanced (T3/4) | 132 (4.7) |
|  | Advanced (N1) | 33 (1.2) |
|  | Missing | 0 |
| Androgen deprivation therapy before inclusion | No | 2,743 (96.8) |
|  | Yes | 90 (3.2) |
|  | Missing | 0 |
| Treatment after inclusion | Radical prostatectomy | 2,403 (84.8) |
|  | Primary radiotherapy | 194 (6.9) |
|  | Radical prostatectomy + adjuvant radiotherapy | 162 (5.7) |
|  | Active surveillance/ watchful waiting | 64 (2.3) |
|  | Missing | 10 (0.4) |
| **Center characteristics** | | |
| Municipality | < 20,000 population | 32 (1.1) |
|  | 20,000–100,000 population | 987 (34.8) |
|  | > 100,000–1,000,000 population | 1,658 (58.5) |
|  | > 1,000,000 population | 156 (5.5) |
|  | Missing | 0 |
| Teaching status | No | 102 (3.6) |
|  | Academic | 2,280 (80.5) |
|  | University | 451 (15.9) |
|  | Missing | 0 |
| Ownership | Nonprofit | 1,459 (51.5) |
|  | Public | 1,269 (44.8) |
|  | Private | 105 (3.7) |
|  | Missing | 0 |
| Primary cases | Continuous | 28 centers  Center mean (SD): 23.22 (33.62)  Center range: 10.2-225 |
| Months since first certification | Continuous | 28 centers  Center mean (SD): 81.65 (37.26)  Center range: 7.6-126.57 |
